# Emulsions Incorporated in Polysaccharide-Based Active Coatings for Fresh and Minimally Processed Vegetables

**DOI:** 10.3390/foods10030665

**Published:** 2021-03-20

**Authors:** Marina Ramos, Cristina Mellinas, Ignacio Solaberrieta, María Carmen Garrigós, Alfonso Jiménez

**Affiliations:** Department of Analytical Chemistry, Nutrition & Food Sciences, University of Alicante, 03080 Alicante, Spain; cristina.mellinas@ua.es (C.M.); solaberrieta@ua.es (I.S.); mc.garrigos@ua.es (M.C.G.); alfjimenez@ua.es (A.J.)

**Keywords:** emulsion, coating, polysaccharides, functional compounds, food shelf-life, food preservation

## Abstract

The consumption of minimally processed fresh vegetables has increased by the consumer’s demand of natural products without synthetic preservatives and colorants. These new consumption behaviors have prompted research on the combination of emulsion techniques and coatings that have traditionally been used by the food industries. This combination brings great potential for improving the quality of fresh-cut fruits and vegetables by allowing the incorporation of natural and multifunctional additives directly into food formulations. These antioxidant, antibacterial, and/or antifungal additives are usually encapsulated at the nano- or micro-scale for their stabilization and protection to make them available by food through the coating. These nano- or micro-emulsions are responsible for the release of the active agents to bring them into direct contact with food to protect it from possible organoleptic degradation. Keeping in mind the widespread applications of micro and nanoemulsions for preserving the quality and safety of fresh vegetables, this review reports the latest works based on emulsion techniques and polysaccharide-based coatings as carriers of active compounds. The technical challenges of micro and nanoemulsion techniques, the potential benefits and drawbacks of their use, the development of polysaccharide-based coatings with natural active additives are considered, since these systems can be used as alternatives to conventional coatings in food formulations.

## 1. Introduction

The current lifestyle of consumers has modified the fruit and vegetable consumption patterns. The request for natural, fresh, healthy, and minimally processed food is continuously increasing mainly due to the desire to consume beneficial health products driven by increased knowledge and awareness of the consumers related to the presence of healthy compounds in these products like antioxidant, antimicrobial or other micronutrients [1]. Minimally processed fruits and vegetables are products that have increased their consumption in a healthy diet. These types of foods have not been physically altered from their native state and remain in a fresh form [2], representing a very promising expansion sector for the food industry [3].

Nevertheless, the consumption of fruits and vegetables is directly related to the product’s freshness and quality when purchased by consumers. Undesirable characteristics, such as color changes, browning, or sweating, could cause rejection and should be avoided, resulting in economic losses, and ethical problems due to the production and accumulation of high concentration of agro-industrial wastes. These changes related to the products freshness and quality can occur during processing operations, such as cutting, washing, or peeling, altering their integrity, modifying their surface properties, and damaging their tissue structure by the presence of undesirable effects, such as enzymatic reactions [2,4]. These degradation processes cause surface browning, undesirable flavor, water loss, and texture collapse, which should be avoided as much as possible. Besides, the presence of microorganisms on the vegetables’ surface could affect the products’ safety and the final quality [5].

Consequently, any solution to reduce these problems is essential to improve the fresh food acceptance, reducing costs, and decreasing economic losses into food processing plants. In this context, the use of edible coatings in fruits and vegetables has been proposed as a valid methodology to improve their overall quality, extending shelf-life, perishability, and increasing profit for growers and marketers. These additives could act as barrier against microbial agents or UV radiation, maintaining or improving the overall quality of foodstuff [6]. In contrast, the application of these thin layers of polymeric materials suitable for human consumption to protect food formulations from external agents can cause several changes in their organoleptic, mechanical, and textural properties [7].

Different approaches have been evaluated to increase food shelf-life and reduce the impact of external agents on their essential properties. In this sense, the combination of biopolymer matrices and emulsified structures based on the encapsulation of functional compounds by dispersion, forming micro- or nano-drops, shows high potential for the formulation of new edible coatings. Some of the research work related to edible bio-coatings focused on improving their properties by exploring the advantages of combining functional compounds, such as vitamins [8], essential oils (EOs) [9], and polyphenols [10,11] with natural polysaccharides like pectin [12], chitosan [13], cellulose [14], or starch [15]. Besides, encapsulation of active compounds resulting in efficient delivery systems requires a complete understanding of how these functional compounds can be encapsulated and their release mechanisms for their application in food formulations [6].

It is well known that different strategies can be used to encapsulate active chemicals, where emulsions show great potential for the food industry. This technique improves the bioavailability of functional compounds, protecting them from environmental storage conditions, and controlling their release into food products [16]. The development of microparticles and nanoparticles using emulsion methods has been widely studied in the last years. Table 1 lists some examples of emulsion systems used in the encapsulation of functional compounds to be further applied to extend shelf-life in vegetable foods.

The present review reports the latest works based on emulsion techniques and their possible application to polysaccharide-based active coatings as advanced carriers of functional substances with antimicrobial, antioxidant, antibrowning, and texture enhancers functionalities for vegetables.

## 2. Emulsion Methods: Microencapsulation and Nanoencapsulation

The emulsion formation consists of the dispersion of liquid drops (dispersed phase) in a non-miscible liquid (continuous phase) and these can be clustered as a function of the droplet size in three groups: Conventional emulsions (drop diameters larger than 1000 nm), nanoemulsions (drop size between 20–1000 nm depending on different authors [23,24,25]), and microemulsions (drop diameters in the 5–100 nm range) [24].

**Figure 1:**
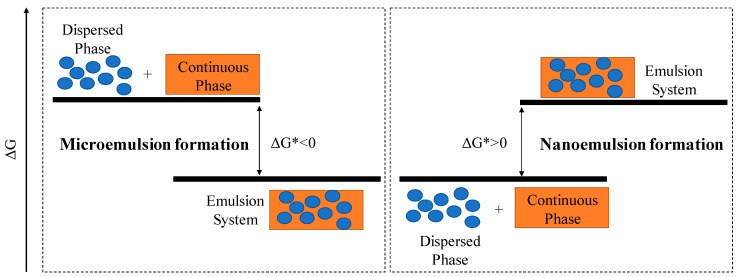
Comparison of the diagrams for the development of microemulsions and nanoemulsions, where ΔG* is the process activation energy.

It is essential to precisely define the difference between microemulsions and nanoemulsions since both types are considered in the same droplet size range (r < 100 nm) depending on the different authors. It should be highlighted that their nature determines the methods used in their preparation, their long-term stability, and functional performance [26]. The main difference between micro and nanoemulsions is their thermodynamic stability, since microemulsions are thermodynamically stable, while this is not the case for nanoemulsions [26,27,28]. Figure 1 shows the comparison of the diagrams of formation of these emulsions based on the activation energy of the process. It can be observed that the free energy of colloidal dispersions is lower than the free energy of the phase separation (continuous and disperse phase), in microemulsions, which translates into higher thermodynamic stability. In contrast, nanoemulsions can be considered as metastable systems because, despite being thermodynamically unstable (ΔG* > 0), they are kinetically stable. However, under certain composition and temperature conditions, nanoemulsions can be maintained stable for a considerable period of time [29,30].

Besides, a third component (surfactant or emulsifier) is needed to develop an emulsion system. Surfactants are amphipathic compounds with a lipophilic part with affinity for the lipid phase, and a hydrophilic part [31]. The role of surfactants is to facilitate and stabilize the formation of drops [32] in emulsions. In turn, surfactants can determine which of the fractions forming the emulsion will be continuous and disperse. A scale based on the hydrophilic-lipophilic balance (HLB) with arbitrary values between 1–20 is used to classify surfactants according to their relative affinity for the oil or aqueous phase. Specifically, a surfactant with oil-like character would show values close to 0, while those with higher affinity for water tend to get high values in HLB [31,33]. Sorbitan esters, commercially known as Span 20, 40, 60, and 80 [34,35], propylene glycol monolaurate [36], distilled monoglycerides [37], glyceryl monostearate [38,39], and lecithin [40,41,42,43] are the most used lipophilic emulsifiers by food industry. The most employed hydrophilic surfactants are proteins or carbohydrates with high molecular weight, such as carrageenan [44], pectin [45], isolated soy protein [46,47,48], or different types of polysorbates, known as Tween 20, 40, 60, and 80 [34,35,49,50,51]. Besides, solid particles, i.e., chitosan nanoparticles [52] or chitin nanocrystals [53], can be also used as emulsifiers producing emulsions known as Pickering-emulsions [54,55,56].

Oil-in-water (O/W) and water-in-oil (W/O) are the most common emulsions, although multiple and mixed emulsions can be obtained. Mixed emulsions are developed by more than one dispersed phase retained by a continuous phase. For instance, a combination of essential oils (EOs) from plants has been developed to study their synergic effect in the antioxidant and/or antimicrobial properties [57,58]. In particular, clove and thyme EOs emulsions incorporated into Farsi gum coatings have been used in refrigerated rainbow trout fillets to increase the shelf-life [57].

The formulation of multiple emulsions results in a system characterized by the coexistence of O/W and W/O emulsions, in which the globules of the dispersed phase contain within them equally dispersed small drops [59]. Multi-emulsions can be classified into oil-in-water-in-oil (O/W/O) or water-in-oil-in-water (W/O/W) emulsions, the latter being the most used by the food industry [60]. The most common method to develop these emulsions consists of two steps: Firstly, a simple emulsion is prepared and then it is dispersed into the suitable continuous phase. In this last step, the applied energy must be lower than energy in the first step since the system formed could be destabilized [61,62,63,64]. These emulsions show great interest by allowing encapsulation and protecting both hydrophilic and hydrophobic bioactive agents [65]. For instance, Huang et al. [63] encapsulated in a W/O/W system, hydrophilic arbutin, and one hydrophobic antioxidant, coumaric acid. The primary emulsion was composed of gelatin, arbutin, and NaCl (to increase the ionic strength of the solution), while the lipid phase contained coumaric acid and polyglycerol polyricinoleate, and it was developed by heating the mixture for 10 min at 55 °C using ultrasounds to improve the emulsion formation. The primary emulsion was further dropped into different protein-polysaccharide complexes to evaluate their functional properties. Another reason for the use of multiple emulsions is the protection of the primary emulsion from environmental changes in humidity, temperature, or pH. In fact, food emulsions stabilized by caseinates are extremely sensitive to destabilization at low pH values, so Estevez et al. [61] developed W/O/W emulsions containing a grape seed phenolic-rich extract encapsulated into sodium caseinate and protected by different polysaccharides, such as carboxymethyl cellulose or gum Arabic. Their results showed that the obtained nanoemulsions were stable at pH range 3–6 when a ternary emulsion was used, and no significant differences were found in the release of antioxidants due to the presence of these hydrophilic emulsifiers.

### 2.1. Microemulsions

**Figure 2:**
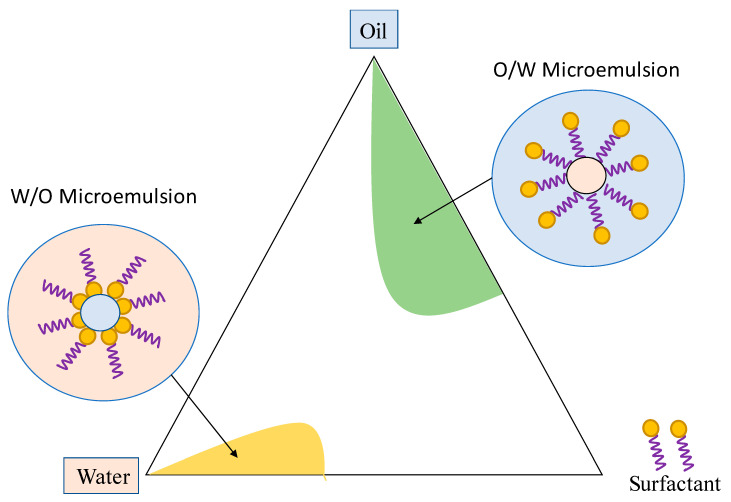
Diagram of a ternary phase of an O/W/surfactant system.

Microemulsions are transparent and thermodynamically stable colloidal solutions in which equivalent amounts of non-miscible liquids can coexist due to the presence of one or more surfactant compounds with a suitable hydrophilic-lipophilic balance (HLB). Microemulsions are usually formed by spontaneous self-assembly of components under appropriate compositions and are easily prepared under mild mechanical mixing [27,28,37,66,67]. In the industrial processing of microemulsions, their thermodynamic stability is a parameter of great interest since the formulation characteristics do not depend on the order of incorporation of the components, the stirring speed, or the cooling kinetics. In general terms, microemulsion properties are maintained over time without coalescence or sedimentation, provided that the same composition and temperatures range for their stability are preserved [26,68,69]. Figure 2 shows an example of a ternary phase diagram of the oil/water/surfactant system. As can be seen, the same system can be obtained either by adding water to an O/W microemulsion or by adding oil to a W/O microemulsion. Besides, between these two extremes, the droplets shape may change depending on the ratio of components, and a bicontinuous system where all components are in equilibrium is obtained [69,70].

Different authors have proposed the development of microemulsions to encapsulate antioxidant and/or antimicrobial agents such as gallic acid [66], apigenin [71], *Oliveria decumbens* essential oil (EO) [72] *Piper betle L*. EO [73], citronella, eucalyptus, and mint EOs [68], thymol [74], or *Eugenia dysenterica* extract rich in catechin [75]. In these microemulsions, all components are mixed to obtain a homogeneous and transparent solution under mechanical stirring. The effect of surfactants and co-surfactants in the successful formation of these microemulsions has been also studied. Results showed that these systems require a high concentration of surfactants (15–30%) [26,30]. In these cases, the oil incorporation capacity was significantly affected by the use of ethanol as a co-surfactant, expanding the area of the ternary phase diagram. For example, the use of different surfactants, and co-surfactants such as Tween 80 and ethanol, has allowed obtaining O/W microemulsions with high stability [76].

### 2.2. Nanoemulsions

Nanoemulsions are usually made up of four components: Oil phase, aqueous phase, surfactant to reduce the interfacial tension, and the external energy provided for their formation, since, unlike microemulsions, these thermodynamically unstable systems require energy to convert the initial components into a colloidal dispersion [30]. The methods to obtain different nanoemulsions can be grouped in two types: High- and low-energy methods. Table 1 shows some examples.

#### 2.2.1. High Energy Methods

High-energy methods are based on the division of large droplets (phase dispersed) into smaller ones by disruptive forces (high shear) in a continuous phase [77]. High-speed homogenization, ultrasounds, and high-pressure homogenization or microfluidization are within this category.

##### High-Speed Homogenization

This method is the most straightforward technique to obtain nanoemulsions with different droplet sizes. In this case, all components of the nanoemulsion are mixed at high speed, i.e., 10.000 [78] or 15.000 rpm [79] for a few minutes. Before mixing, all components of each phase are mixed separately [78]. Sometimes, the addition of the dispersed phase is carried out dropwise onto the continuous phase by gentle agitation before applying high speed [79].

The concentration of different phases affects significantly the nanoemulsion droplet size. For instance, Mendes et al. [15] evaluated the droplet size, polydispersity index (PDI), and morphology of a pectin-based nanoemulsion when the concentrations of pectin and the lemongrass EO were changed. Results showed that the droplet size increased at high pectin concentrations due to the presence of attractive forces between the different pectin molecules. In contrast, when the concentration of the EO increased, the droplet size of the nanoemulsion decreased due to the presence of amphiphilic compounds in the EO, which may contribute to droplet size reduction.

In contrast, the combination of high-speed homogenization with other techniques has shown great potential to produce nanoemulsions with small droplet size and low PDI. The objective of these strategies is to form a primary emulsion, which is further subjected to a size reduction process using high-pressure [35,51] or ultrasounds [49].

##### High-Pressure Homogenization

This is the most popular technique to obtain nanoemulsions at the manufacturing scale. In this method, a primary emulsion is used, generally prepared by using a high-speed mixer. This emulsion is further passed through the high-pressure homogenizer, where it undergoes a combination of intense shear and cavitation, causing large droplets to break down resulting in smaller ones. Pressure and number of cycles in the homogenizer have a significant effect on the particle size [23,24,80]. Cordoba et al. [35] evaluated the effect of pressure on the droplet size using a range between 69–100 MPa. Results showed a clear reduction in droplets size when the pressure increased. Besides, the composition of active compounds present in nanoemulsions also affects the droplet size and PDI. The same conclusions were obtained by Alexandre et al. [51] when developing nanoemulsions using a high-pressure homogenization method with ginger EO at different concentrations.

##### Ultrasounds

The mechanism of generating a nanoemulsion using ultrasounds is attributed to the cavitation of bubbles. During this process, ultrasounds cause the formation, growth, and collapse of microscopic vapor bubbles within the liquid. When these cavities collapse, energy is released, which is enough to increase the droplets’ surface area decreasing their size [81,82]. Different EOs have been encapsulated using ultrasounds, including *Zataria Multiflora* [45,53] and *Marjorama* [9] EOs. For this purpose, different irradiation times were tested, but, the most common strategies for the ultrasounds synthesis of nanoemulsions involve short times (0–30 min) [40,50,83]. The application of ultrasounds with different pulses has been also investigated. This approach has shown great potential in temperature-sensitive compounds since it avoids overheating [58,83]. Regarding experimental conditions, power or amplitude of the ultrasound waves could affect the nanoemulsions’ droplet size. In general terms, when these variables increase, the droplet size decreases while the system’s temperature is higher [50,83].

#### 2.2.2. Low-Energy Methods

Low-energy methods can be explained by the modification of the system’s state by manipulating some of the formulation variables. The objective in this approach is to modify phases in such a way to produce changes, reducing the droplet size [25]. Some of the most important low-energy methods are phase inversion (point inversion temperature (PIT) and emulsion inversion point (EIP)), as well as spontaneous emulsification.

##### Phase Inversion Methods

The PIT method can describe changes in the system temperature by rapid cooling to force nanoemulsion networks to break-up. In general terms, the mixture is heated and then cooled causing a change in the surfactant’s affinity that causes the spontaneous formation of small droplets. The variation in the surfactant’s affinity is a consequence of a change in solubility due to the dehydration of its head group [25,65]. When the phase inversion occurs, the system shows low surface tension, so it is necessary to quickly cool the mixture to keep the obtained nanoemulsion stability [84]. Chuesiang et al. [65] mixed cinnamon EO with Tween 80 surfactant for 30 min at 25 °C to obtain a good nanoemulsion using the PIT method. Then, they heated the mixture to 67–78 °C (depending on the inversion temperature), and finally, they rapidly cooled the obtained emulsion to 4 °C in two steps. Besides, they studied the influence of Tween 80 on the droplet size. Results showed that the increment of the surfactant concentration produced a clear reduction in the droplet size due to the low interfacial tension at the oil-water interface, promoting formation of small droplets when the system was cooled.

The EIP method is based on a change in the system composition. Starting from a primary emulsion, the dispersed phase concentration is changed, causing the surfactant to move towards that phase, generating the system inversion [33]. For example, for a primary W/O emulsion, water is added to the system to form an O/W nanoemulsion. Nanoemulsions containing different antioxidants, such as quercentin [85] or curcumin [86], have been developed. For this purpose, the aqueous phase was added using a peristaltic pump to control the phase inversion process.

##### Spontaneous Emulsification

Spontaneous emulsification occurs through different mechanisms that involve diffusion, thermal fluctuation, and ultra-low interfacial tension. This method is recognized as self-emulsification. In this method, the chemical energy released due to a dilution process in the continuous phase can be used, generally at a constant temperature, without any phase transition [25,87]. The composition of each phase has a significant role in the droplet size. As exhibited in Table 2, the same experimental conditions applied to different matrices resulted in different droplet size [88,89]. These authors worked with the same aqueous phase (Tween 80 and distilled water), but their oil phase was different. Liew et al. [88] used the lime EO and corn oil, and their nanoemulsions had a droplet size between 21–60 nm, while Yildirim et al. [89] developed their nanoemulsions with coconut and cinnamon oils at different concentrations with a droplet size between 81–343 nm.

## 3. Polysaccharides and Functional Properties to Form Emulsion Based Coatings

Dipping, spreading, and spraying treatments have been widely used for the application of coatings onto food surfaces. In addition, volatile and non-volatile solutions of antimicrobial or antioxidant agents can be used to extend the products shelf life by avoiding microbial growth and enzymatic degradation. However, many authors have reported that it is necessary the protection of these compounds to avoid problems in the addition to food formulations, such as their poor solubility, uncontrolled release, high dosages, degradability, etc. [1].

Thus, microemulsions and nanoemulsions could be considered as and innovative and promising strategy to protect, encapsulate, and control the release of active compounds into food formulations. Furthermore, these techniques in combination with edible coatings could enhance the functionality of the overall systems.

**Figure 3:**
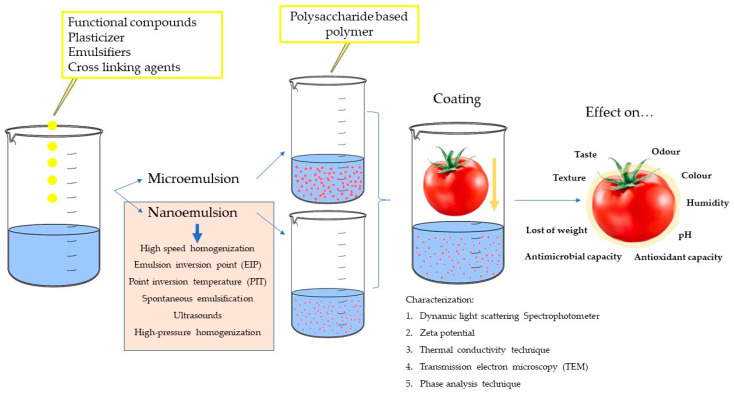
Formation of emulsion-based coatings and effects on fresh food (adapted from [115]).

Figure 3 depicts the formation of emulsion-based coatings and their effects on fresh food. Other compounds, such as plasticizers, surfactants, and cross-linking agents, are also necessary to improve the emulsions functionality. Different techniques can be used to characterize the coatings final properties as well as to transform the physical and functional characteristics of the final coating.

The biopolymers used to obtain the coatings are achieved from abundant, renewable, and low-cost natural sources, while agricultural by-products and wastes have been gaining major importance in the last decades as unprocessed resources for the biopolymers production [91,92]. Among them, polysaccharides (PS) are a wide group of compounds with varied qualities and functional properties. PS like cellulose, chitosan, pectin, alginate, and their combinations have been extensively employed in formulations such as films and coatings intended for food packaging [45,93,94,95,96]. The main advantage of these materials is their biodegradability, biocompatibility, and susceptibility towards biochemical modifications. Generally, PS structures exhibit intrinsic good mechanical and barrier properties against O_2_ and CO_2_ [97]. However, owing to their hydrophilic nature, the low water vapor barrier properties represent a major drawback for these applications. In this sense, great effort has been made to improve this property to obtain biomaterials with good barrier and mechanical properties that could replace synthetic polymers [6]. Some strategies have been tested so far, including biopolymer modification or functionalization [98], development of composite or multilayer materials [99,100], and emulsification with lipophilic compounds [82], among others.

### 3.1. Polysaccharides as Based Polymers

#### 3.1.1. Cellulose

Cellulose is one of the most abundant polysaccharide in nature. It could be isolated from many sources such as wood, plant-based materials, and agricultural by-products, and it can be also produced by microorganisms. This low-cost, non-toxic, biocompatible, biodegradable, and chemically stable linear polymer is an important unprocessed substance for edible films, biodegradable, and coating formulations. However, the low capacity to be assimilated by humans as well as low solubility limit the application of cellulose for food packaging [96]. In this regard, new families of modified-cellulose have been developed to overcome these significant drawbacks [92]. Cellulose derivatives like methylcellulose (MC), hydroxypropylmethylcellulose (HPMC), and carboxymethylcellulose (CMC) could be obtained following a chemical process where native cellulose is used, followed by a reaction with chloroacetic acid, methyl chloride, or propylene oxide [97]. These cellulose products are extremely water-soluble and they are often found in edible films and coating formulations, producing odorless, tasteless, and transparent materials [101,102]. However, it should be noted that this high solubility in water limits their application in coatings to be used in high relative humidity environments [98]. In this sense, some authors have suggested the combination of hydrophobic materials, such as wax, lipids, or fatty acids, into the film or coating-forming solutions to improve barrier to water vapor. In contrast, it has been reported that bacterial cellulose exhibits crystallinity, above average mechanical strength and hydrophilicity than the plant cellulose counterparts [103].

#### 3.1.2. Chitosan

Chitin is the second most abundant biopolymer on nature and it is found in the exoskeleton of shellfish and some insects and cell walls of fungi and yeast. This PS is converted into chitosan by removing acetyl groups through alkaline or enzymatic deacetylation [97], depending on several factors such as time, temperature, atmosphere, alkali concentration, and chitin natural source, among others. Variations in these factors lead to a wide variety of commercial chitosan-based products with different deacetylation degree, molecular weight, and characteristics, which could be used to obtain materials with different properties [96]. Chitosan is considered the only natural PS with intrinsic antimicrobial activity against bacteria, fungi, and yeasts [97,104]. Besides, it has been reported that chitosan and derivatives possess other interesting functionalities, including antioxidant, anticancer, and anti-inflammatory effects, among others [105]. Furthermore, chitosan is classified as *Generally Recognized as Safe* (GRAS) by the United States Food and Drug Administration (USFDA), since it is non-toxic, biodegradable, and biocompatible. It has also good film-forming properties and has been extensively reported in the development of coatings for active food packaging. Chitosan-based films could be produced by different techniques, such as direct casting, coating, extrusion, or layer-by-layer assemblies. On the other hand, several food preservatives, EOs, and other biopolymers have been successfully incorporated into chitosan-based materials [104,105,106].

#### 3.1.3. Alginate

Alginate is a biodegradable, biocompatible, non-toxic, and low-cost linear co-polymer commonly produced from several seaweed species [107] with a molecular structure comprising alternating α-*L*-guluronic acid and β-D-mannurocin acid units linked by 1–4 glycosidic bonds. The polymeric structure depends on the alginate source and directly affects its physical and chemical properties [95,107]. Alginate has been also classified as a GRAS substance and it is currently utilized in many food applications, including food packaging formulations for vegetables, meat, and seafood products. As a result of its good film-forming properties, several methods such as solvent casting, extrusion, and coating application have been used in the production of alginate-based films, A wide variety of antimicrobial, antioxidant, flavoring, antibrowning, and nutritional agents have been included into alginate-based active formulations for vegetables and fruits, meat and sea products, and cheese [95].

#### 3.1.4. Pectin

Pectin is found in plant cell walls and intercellular layers of all land plants and it is a natural biopolymer. It is principally produced from citrus and apple peels, showing pectin from lemon and lime peels the highest quality and capacity to isolate in pure fractions. Regarding structure, homogalacturonan is the major component of pectin-based polysaccharides. However, hamnogalacturonan-I and rhamnogalacturonan-II are part of their composition [97]. The pectin source, extraction methods, and their conditions directly affect the degree of esterification, the molecular weight, and aceylesterification process. Pectin has been extensively employed as thickening, stabilizing, and gelling agent in food products [93].

#### 3.1.5. Gums

Gums are polymers of naturally occurring PS with the capability to hydrate in water either by forming a stabilizing emulsions or gel. They could be classified according to their origin (i.e., plants, seeds, microbial exudates, seaweed, among others), shape (i.e., short branch or multiple-branched structures), charge (i.e., non-ionic or anionic gums), and chemical structure (i.e., galactomannans, glucomannans, uronic acid, among others). Gums have been classified as GRAS and their use is safe for food applications. For instance, they have been used as food stabilizers, thickeners, gelling agents, as well as in food packaging applications [108]. Besides, they have been widely used in coatings providing a semipermeable barrier to reduce weight loss and respiration rate, while maintaining nutritional value, and enhancing sensory properties such as the overall appearance, color, texture, or flavor, resulting in the extension of the product shelf life. Moreover, gums have been successfully used as carriers of active substances such as essential oils, plant extracts, and phenolic compounds, to enhance the mechanical barrier, antibrowning, antioxidant, and antimicrobial properties of coatings [109,110].

### 3.2. Methods for the Application of Coatings

Dipping, spreading, and spraying are well-recognized methods to be used for the application of edible coatings by the food industry.

Spraying is widely used by the small droplet size (around 20 μm) resulting in very low solutions viscosity, which facilitates their efficient application into food. This technique works at high pressure, and the products need to be turned upside-down for the uniform application of the coating onto the entire surface of the product [111]. The coating homogeneity also depends on the drying time, temperature, and the application equipment. Alginate-based edible coatings with carvacrol and methyl cinnamate sprayed on fresh strawberries increased shelf life and antioxidant capacity [112].

Spreading is a handy and straightforward method that is often used for high viscosity coating solutions. However, in some cases, the coatings formation could be non-uniform. Dipping is also very useful for the production of food coatings. In this technique, food is totally submerged in the coating solution generally for 5–30 s, creating a thin coating layer on the surface of the product, followed by drying at room temperature [113]. The main disadvantage of this technique is based on the presence of anaerobic conditions if the thickness of the film formed is not adequately controlled [114].

Panning is another method used in food and cosmetics. Samples are coated by oscillation to achieve a perfect and homogeneous coating. Once coated, they are dried in a forced-air stream or by increasing the temperature.

### 3.3. Effect of Emulsion on Polysaccharide-Based Coatings.

Much research has been developed in the last years to develop new packaging strategies to increase the food products’ shelf life, and in particular active edible emulsion-based films and coatings have attracted much attention. In this sense, a wide variety of antioxidants, antimicrobials, flavors and aromas, vitamins, and probiotic agents have been incorporated to different PS-based matrices to improve their properties (Figure 3).

The emulsion is used as an encapsulation technique, and the biopolymer is used as a support for these encapsulations and as a coating on fresh vegetables. In this sense, during the formation of the film, numerous mechanisms such as intermolecular bonding, covalent unions, and electrostatic, hydrophobic, and ionic interactions between polymer chains and various functional ingredients can be presented [116].

Cellulose derivatives have been used as food preservatives or essential oil carriers to develop edible active coatings for minimally processed or fresh vegetables. In this sense, edible coatings based on hydroxypropyl methylcellulose (HPMC) with beeswax (BW), and food preservatives were evaluated on cherry tomatoes stored under refrigeration [117]. In this case, emulsions were prepared using a high-shear probe mixer to homogenize an aqueous HPMC solution (5 wt%) with the beeswax and the corresponding food preservative (2 wt%). In all cases, glycerol, oleic acid, and Tween 80 were employed as emulsifier, plasticizer, and surfactant, respectively. Stable formulations with no phase separation and a viscosity range of 100–150 cps were obtained. It was reported that the moisture and gas barrier of these coatings were influenced by the active principle integrated into the emulsion. Indeed, the coating with ammonium carbonate decreased the weight loss, and those formulated with both ammonium salts (ammonium phosphate and ammonium carbonate) reduced the respiration rate of the packaged food. The physicochemical properties and sensory quality of cherry tomatoes were maintained in all cases, and all coatings drastically decreased the *Botrytis cinerea* proliferation.

On the other hand, HPMC with oregano and bergamot EOs was employed in the formulation of an edible coating for fresh plum packaging [118]. In this case, emulsions were obtained by using mechanical overhead stirring, mixing an HPMC aqueous suspension (4%, *w/v*) with each EO (2 wt%) at room temperature. Results suggested that incorporating EOs with the emulsion formulations did not change the thickness of the obtained films. Due to the intrinsic hydrophobicity of the EOs, the moisture content of the HPMC films diminished considerably, and the contact angle of water on the outer surface of these films were considerably enhanced with the EO concentration. The combination of EOs with HPMC coating formulations resulted in lower transparency, higher oxygen transmission rates, and no apparent changes in the water vapor barrier properties compared with a pure HPMC film. Moreover, both coating treatments demonstrated considerable antimicrobial effect against the growth of microbial cells resulting in promising approaches for expanding the shelf life of plums during storage.

Carboxymethyl cellulose (CMC) is another cellulose derivative that has been used in active coating formulations in fresh vegetables. As an example, pears were coated using a CMC, candelilla wax, and potassium sorbate emulsion to prevent fungal infection under simulated retail conditions [119]. In this case, the CMC aqueous solution (5 wt%) containing sorbitol (3 wt%), candelilla wax (0.5 wt%), Tween 40 (0.35 wt%), and potassium sorbate (3 wt%) was emulsified in a homogenizer and the resulting films showed high effectiveness to control fungal growth. However, the coated fruits showed much higher weight loss than the uncoated counterparts. This unexpected result in coated films was explained by their enhanced gas barrier properties, which modified the internal atmosphere in plant tissues leading to higher respiration rates and, consequently, higher weight losses. Thus, this CMC-based coating failed to extend pears shelf-life since it also induced losses in appearance quality of the product.

Chitosan is another PS that has been widely used in active coating formulations. Edible coatings based on CMC and chitosan incorporating citral nanoemulsions were applied to fresh-cut melons to prevent their microbial deterioration and extend shelf-life [113]. In this case, three different citral coarse emulsions (0.8, 1.6, and 2.4% *v/v*) were prepared by homogenization with sunflower oil, Tween 80, and distilled water. These emulsions were further ultra-sonicated to obtain nanoemulsions. Then, CMC and chitosan coating solutions (1.5% *w/v*) were mixed with citral nanoemulsions at three parts of the polysaccharides solution to two parts of the nanoemulsion and melon pieces were coated by dipping into the resulting mixture. Dynamic light scattering and light microscopy shown considerable changes in the droplet size of coarse and nanoemulsion formulations. Furthermore, nanoemulsions showed high stability with no phase separation for weeks at room temperature. Interestingly, these authors found that the addition to a polysaccharide matrix increased the stability of the citral-sunflower oil emulsions. Besides, nanoemulsion coatings resulted in higher water vapor barrier, better mechanical properties, improved appearance, superior antimicrobial protection, and extension of the product shelf-life than the coarse emulsion counterparts.

A similar edible coating was tested on grape berries against Salmonella typhimurium [120]. In this case, emulsions based on chitosan-lemongrass EO were obtained by high shear mixing (HSM) and dynamic high-pressure processing (DHP), with further use of the dipping technique to coat grapes. Here, the particle size of the DHP emulsions was significantly lower than HSM emulsions, leading to more stable emulsions with higher activity against *Salmonella typhimurium,* mesophilic aerobes, yeasts, and molds, while also showing antioxidant performance. The DHP emulsion coating contributed to minimize variations in the sensory characteristics related to the uncoated fruits.

Similarly, chitosan coatings containing lemon EO were used to control the fungal proliferation on strawberries [121]. In this case, two different emulsions were produced by using simple homogenization and homogenization followed by microfluidization, respectively. These differences in the production of the emulsion-based coatings resulted in changes in the volatiles profile and the fruits metabolic pathways. Authors found that pure chitosan coatings lead to the formation of esters and coatings containing the lemon EO, and it incorporated terpenes to their volatiles profile, and enhanced the fermentative processes modifying the typical fruit aroma. Moreover, the addition of lemon EOs to chitosan-based coatings promoted a delay in ripening and succeeded in preventing fungal decay, especially in samples coated with formulations obtained by the two-step homogenization. However, their application is even restricted for their adverse effect on the strawberries aroma.

On the other hand, the inhibition of *Penicillium italicum growth* on chitosan-coated oranges was reported [122]. Chitosan solutions (1% *w/v*) were emulsified at room temperature, including bergamot, thyme, or tea tree EOs (2 wt%) by using a rotor-stator homogenizer, and further curative and preventive coating treatments. The addition of EOs to chitosan formulations caused in slight improvement of the water barrier properties, as expected. In fact, several studies reported the enhancement of the water barrier of polysaccharide coatings by the incorporation of non-polar substances [123]. In this case, chitosan coatings containing the tea tree EO showed the most remarkable antifungal effectiveness for non-damaged fruits (i.e., preventive treatment), whereas the thyme EO-chitosan coatings resulted more effective for previously damaged fruits (i.e., curative treatment). The application of these coatings did not influence significantly on the product respiration during the storage time.

Carvacrol, cinnamaldehyde, and *trans*-cinnamaldehyde have been incorporated in emulsion-based chitosan coatings to preserve quality and safety during post-harvest storage of blueberries [124]. In this case, chitosan coating solutions (1% *w/v*) containing glycerol (0.75% *v/v*), Tween 20 (0.25% *v/v*), and essential oils (0.1 and 0.5% *w/v*) were obtained. Authors found that these coatings reduced the moisture and firmness losses and prevented softening, extending the product shelf-life. Besides, carvacrol and trans-cinnamaldehyde incorporated these emulsions with improved antimicrobial activity.

The antimicrobial activity of modified chitosan and mandarin EO nanoemulsions with non-thermal treatments on green beans were demonstrated against *Listeria innocua* (ozonated water, UV radiation, and γ-irradiation) [125]. In this case, a mixture of sunflower oil, glycerol, and 0.05% (*w/v*) mandarin EO was dispersed in distilled water containing Tween 20 by using an Ultra Turrax T25 stirrer. The nanoemulsion was further obtained by submitting this initial emulsion to high-pressure homogenization to get modified chitosan solutions (1% *w/v*). Authors concluded that this approach was effective in controlling the growth of *Listeria innocua* on vegetables, but changing significantly some physical properties, such as color and firmness.

Similar research was performed to estimate the effectiveness of a carvacrol-modified chitosan nanoemulsion coating combined with modified atmosphere packaging and γ-irradiation against *Escherichia coli* and *Salmonella Typhimurium* on green beans [126]. A mixture of sunflower oil, glycerol, and carvacrol was dispersed in distilled with Tween 20 using an Ultra Turrax T25 stirrer. The nanoemulsion was obtained by submitting this primary emulsion to 5 cycles of high-pressure homogenization and it was incorporated at 0.05% (*w/v*) into modified chitosan solutions (1% *w/v*) mixed vigorously with an Ultraturrax stirrer. Authors found that a combined treatment of γ-irradiation, functional coating, and modified atmosphere packaging was extremely effective against these pathogens.

Other authors studied the combination of carvacrol nanoemulsions and modified chitosan active coatings with pulsed light treatments against *Escherichia coli* on cucumber slices [127]. In this case, carvacrol nanoemulsions were formulated by high-pressure homogenization and then incorporated at different concentration levels (0.03 and 0.08 wt%) by Ultraturrax mixing into modified chitosan solutions (2 wt%). Cucumber slices were dipped into these solutions and a synergic effect was observed when both treatments were combined, reaching > 5 log cycles of *Escherichia coli* reduction.

In recent years, several alginate-based films for coatings were developed [107,112,128,129]. Alginate-based coatings containing lemongrass EO nanoemulsions at several concentration levels were tested on *Fuji* apples against *Escherichia coli*, psychrophilic bacteria, molds, and yeasts [128]. Sodium alginate solutions (2% *w/v*) were mixed with the lemongrass EO forming coarse or nanoemulsions (0.1, 0.5, and 1% *v/v*) and cut *Fuji* apples were dipped into them. Results showed that nanoemulsions exhibited better performance than conventional emulsions, and all tested lemongrass EO concentrations were useful in inactivating *Escherichia coli strains*. However, high concentrations of the lemongrass EO resulted in surface browning in apple pieces, raising the conclusion that concentrations higher than 0.1% (*v/v*) were detrimental for the fruit integrity and shelf life. A clear increase in nanoparticles number was observed when using a low EO concentration in the formulation. The droplet size of emulsions incorporated into edible coatings did not significantly influence the quality parameters such as firmness and color during storage in this case.

Pectin has been widely used in edible active coatings. In this sense, the effect of pectin-based edible coatings incorporating nisin as an active principle, and combined with modified atmosphere packaging, was studied against enzymatic browning, *Escherichia coli, Salmonella enteritidis, and Listeria monocytogenes* on fresh-cut persimmon [130]. Pectin solutions were emulsified with oleic acid and Tween 80, glycerol was added as plasticizer, and calcium chloride and citric acid were used as antibrowning agents. Cut persimmon pieces were dipped into coating emulsions and stored 9 days at 5 °C. Authors concluded that the application of the active edible coating combined with modified atmosphere packaging was a very effective treatment to prevent enzymatic browning while also reducing the microbial proliferation during storage, extending the shelf life, and preserving the visual quality persimmon slices.

## 4. Application of Emulsion Techniques to Vegetables

Vegetables are known by their nutritional benefits since they provide antigenotoxic, anti-inflammatory, antioxidant, anti-allergic, anticancer, and anti-diabetic functions. The new trends focusing on the need of a healthy diet has propelled the consumption of fresh-cut and ready-to-eat vegetables and fruits, changing the traditional consumption patterns of these food products.

It is well known that the processing operations (i.e., washing, cutting, or peeling) can produce alterations in food by modifying the vegetables final appearance and decreasing their nutritional value. In some of these operations, the outer layers of vegetables cells are damaged and, on many occasions, they are the trigger of enzymatic reactions, which are mostly detrimental for food. Some authors described these modifications as changes in surface by browning, unexpected flavor, water loss, and texture breakdown [131]. Besides, organoleptic degradation effects due to the growing of microorganisms onto the vegetables surface are observed and they must be eliminated or limited since they cause consumer´s rejection and represent a risk for health. In summary, these limitations in processed vegetables should be minimized. With this background, the use of coatings can be considered a valid alternative to incorporate active substances to protect the food structure and their resistance against aggressive external factors while increasing shelf life, decreasing production costs, and improving consumer acceptance [115].

One of the most explored options for the utilization of coatings in fresh-cut and ready-to-eat vegetables is focused on the development of the film layer directly onto the product surface by spraying or dipping the coating solution formed by an edible film containing the encapsulated active principles [4,132]. Table 3 summarizes the most relevant works published in the last decade, related to the use of polysaccharides to obtain edible coating films with functional properties to extend their shelf-life and retain the quality of vegetables.

Robledo et al. [83] evaluated the influence of thymol nanoemulsions incorporated to quinoa protein-chitosan coatings on the mold growth in inoculated cherry tomatoes. These authors used two different methods to obtain nanoemulsions, the spontaneous method, and ultrasounds. In both cases, the droplet diameter was lower than 200 nm. However, the nanoemulsion obtained by applying the spontaneous method showed better antimicrobial activity and lower particle hydrodynamic diameter (0.20 ± 0.01 nm) than nanoemulsions obtained by ultrasound-assisted methods. The application of these coatings to cherry tomatoes inoculated with *Botrytis cinerea* showed a significant decrease of the yeasts and molds counts recorded after 7 days. The coating with the nanoemulsion showed a population around 4.7 log CFU/g with the control around 6.28–6.45 log CFU/g, indicating the antimicrobial activity of these nanoemulsion-based coatings. Mustafa et al. [134] used a chitosan-surfactant nanostructure with micelle size between 400 and 800 nm to coat tomatoes. Some quality parameters, such as texture, flavor, nutritional quality, appearance, and safety, were monitored using different analytical methods. These chitosan-based coatings showed their good performance in delaying deterioration, extending shelf life and subsequent post-harvest losses in tomatoes. For example, ripening was delayed 5 days showing no modifications on texture, firmness and color. Cherry tomatoes were superficially inoculated with a *Botrytis cinerea* spore suspension previously to immersion in the coating solution based on hydroxypropyl methylcellulose, beeswax, and food preservatives (potassium carbonate, sodium propionate, ammonium carbonate, and ammonium phosphate) [117]. After 14 days at 5 °C, followed by 7 days at 20 °C, the authors concluded that these active coatings significantly reduced the *Botrytis cinerea* proliferation, with sodium propionate the most effective against this pathogen and ammonium carbonate the most effective controlling weight loss and maintaining the firmness of cherry tomatoes.

Carrot is a common root vegetable known by being an important source of β-carotene, necessary for synthesizing Vitamin A, minerals, and some phenolic antioxidants. However, when submitted to post-harvesting processes, carrots suffer organoleptic deterioration showing undesired changes in color, texture, and odor. The whiteness index is a parameter that can relate to quality deterioration in fresh-cut carrots products. This effect is named cut surface whitening/white blush, and it is due to surface dehydration and lignification of wounded tissues. Ranjitha et al. [136] analyzed the effects of several chemical agents in coatings to be used with cut carrot. Calcium chloride, calcium propionate, chlorine, chitosan, sesame oil emulsion, hydrogen peroxide, ascorbic acid, and a mixture of ascorbic acid and ethylenediamine tetraacetic acid (EDTA) were tested with low methoxy pectin and two more polymers and the total phenolic content and the flavonoids profile were determined. Results indicated that the presence of lignin precursors, such as vanillic acid, coumaric acid, and myricetin, suggests a substantial reduction in cut surface blanching, astringent and bitter taste in the active pectin coatings. Besides, the results in the antimicrobial activity against aerobic bacteria recorded after 12 days of storage were lower than threshold values established by the Spanish legislation for microbial populations for safe consumption in minimally fresh-processed fruits which is 7 log CFU/g.

Ben-Fadhel et al. [138] used natural antimicrobials such as those present in a mixture of four EOs and citrus extract extracted from different parts of *Cinnamomum verrum,* *Cymbopogon winterianus, Cymbopogon flexuosus*, and *Origanum compactum*. This trend is increasingly widespread since these compounds can reduce the antimicrobial activity or even eliminate microorganisms, like pathogenic bacteria, yeasts, and molds, increasing the shelf life and quality of foods. The oil-in-water emulsion obtained by these authors was composed of a mix of the citrus extract and a mixture of the EOs with sunflower lecithin and sucrose monopalmitate as emulsifiers. The cut carrots were dipped in the antimicrobial coating solution constituted by pectin with the oil-in-water emulsion. The results showed that pectin’s presence could stabilize the bioactive compounds releasing them in a controlled way. Regarding the antimicrobial properties, a significant inhibition on the growth of *Listeria monocytogenes* was obtained, showing the same inhibition at day 8 and 15 for the pectin-based coatings, (around 2.2 log CFU/g); with the growth in the control 8 log CFU/g after 15 days [138]. Martínez-Hernández et al. [137] also used carvacrol added to a chitosan matrix to stabilize the nanoemulsion used as coating in cut-carrots. The nanoemulsion was obtained by introducing carvacrol dropwise into the aqueous chitosan solution with homogenization with an Ultra-Turrax stirrer. The nanoemulsion managed to trap the undesired flavor of carvacrol, showing good organoleptic acceptability with no off-flavors, while the carrot slices coated with the active formulation showed reduction in whitening after 13 days of storage. Besides, considering that carvacrol is known for its antimicrobial activity [149], it was observed that these active coatings can reduce microbial growth during the first nine days of storage. It was also noticed that these active coatings can control microbial growth while maintaining low values of lactic acid bacteria, yeasts and molds and mesophiles, psychrophiles, and *Enterobacteriaceae* when compared to control samples [137].

The effects of bioactive coatings on fresh cauliflowers quality were evaluated by Afia Boumail et al. [140]. They used citrus extract, lactic acid, and lemongrass EO as antimicrobial agents and maltodextrin, starch, and methylcellulose as the polymer matrix to prepare the coating. These authors also looked for synergies between the antimicrobial properties of the emulsions combined with gamma irradiation and negative air ionization with ozone [139]. The combination of nanoemulsions with non-thermal treatments has also been evaluated in coatings for green peppers. Maherani et al. [22] studied the combination of antimicrobial nanoemulsions and non-thermal treatments (irradiation with gamma rays, X-rays, or electrons) to offer advantages to pre-packaged or bulk foodstuff, by decreasing pathogenic food bacteria that can cause food harming, controlling insect plagues, delaying the fruit ripening, and preventing vegetables from germination. In this sense, the combination of non-thermal treatments with active nanoemulsions could be applied to prolong their shelf-life by preserving firmness, sensory attributes, and color.

A modified chitosan-based coating combining one nanoemulsion of EOs with gamma irradiation was also proposed by Severino et al. [126] to extend the shelf-life of green beans. Four different active compounds were used: Mandarin, bergamot, lemon, and carvacrol EOs in chitosan-based coatings. The carvacrol nanoemulsion was most effective against *Salmonella Typhimurium* and *Escherichia coli* O157:H7. Chitosan with the mandarin EO nanoemulsion was used as coating in green beans to evaluate the impact on color, texture, and microbial proliferation during refrigerated storage at 4 °C [125]. Similar results in the protection of green beans were obtained by Donsì et al. [135] when chitosan with mandarin EO nanoemulsion and coupled to some non-thermal treatments.

Cucumber also shows short shelf-life in the natural state, mainly due to the fungal decay in just days. Several strategies have been developed and applied to vegetable samples to extend their shelf-life. For example, Moalemiyan et al. [150] used pectin-based emulsions to preserve cucumbers, preserving their quality, and extending their post-harvest shelf-life. On the other hand, Maleki et al. [7] evaluated the quality-related properties of immature cucumber stored at different temperatures (4, 10, 20 °C) using modified atmosphere packaging and chitosan-based coatings with a limonene emulsion. The fungal decay was evaluated, and authors observed a synergic effect between chitosan and limonene due to both compounds present antimicrobial activity against a broad spectrum of microorganisms. Other authors studied the combination of modified chitosan active films loaded with carvacrol nanoemulsions with pulsed light treatment against *Escherichia coli* on cucumber slices [127]. In this case, modified chitosan dissolutions (2 wt%) were loaded with the carvacrol nanoemulsion at different concentration levels (0.03 and 0.08 wt%) to be used with cut-cucumber slices. A synergic effect was observed when both treatments were combined, reaching >5 log cycles of *Escherichia coli* reduction.

Emulsion-based edible and active chitosan coatings with lemongrass EO were applied to bell pepper fruits to control anthracnose [141]. Lemongrass EO (0.5% and 1.0% *v/v*) was added to chitosan solutions (0.5 and 1.0 wt%), and bell pepper fruits, previously inoculated with 10 µL of *Colletotrichum capsici* spore suspension, were coated by dipping. After 21 days of incubation at room temperature, authors concluded that this coating was effective against anthracnose, improving the fruit quality.

## 5. Conclusions

This review has shown and discussed some of the emulsion methods, i.e., microencapsulation and nanoencapsulation that can be used in the formulation of food coatings. A variety of methods have been developed to obtain different emulsions, forming excellent biomaterials to be applied as edible films in combination with biopolymer matrices. The significance and widespread applications of edible films to obtain coatings to protect food are originated from their unique properties, such as biocompatibility, biodegradability, and no toxicity.

The encapsulation of functional substances, such as EO, by forming emulsions to maintain fresh-cut vegetables and fruits by forming coating systems in the food industry is owed to their particular and advantageous properties, such as:

- Antimicrobial activity: Vegetables coated with active emulsions have been proven to be less sensitive to microbial infection and proliferation than undamaged vegetables, resulting in increasing shelf-life and quality of minimally processed vegetables and fruits.
- Antioxidant performance to avoid or limit the lipid oxidation and quality parameters as appearance and color.
- Film-forming systems help to preserve cell wall integrity and the texture during storage to avoid enzymatic degradation and rejection by the consumers

Much research has been performed in the last few years and much more is currently underway to explore possible commercial applications of polysaccharide-based coatings with functional properties in minimally processed vegetables and fruits, but most of it has been carried out at the laboratory scale. However, the next step to get final commercial applications of these active coatings is still pending, and scale-up and industrial trials at a bulk volume should be carried out in the next future.

## Figures and Tables

**Table 1:** Some emulsion systems applied to vegetables.

Emulsion Technique *	Functional Compounds	Benefits	Food	Ref.
**Ionic gelation technique**	Cuminum cyminum EO	Antimicrobial activity.	Mushroom	[17]
**--**	*Trans*-cinnamic acid	Antimicrobial and antibiofilm effects.	Lettuce	[18]
**Ultrasonication**	Oregano EO	Antimicrobial activity.	Lettuce	[19]
**HPH**	Carvacrol	Antimicrobial activity.	Zucchini	[20]
**HPH or ultrasonication**	Carvacrol	Antimicrobial activity.	Shredded cabbages	[21]
**HPH**	Lemongrass EO Citrus extract Fermented dextrose Prolong 2 Concentrated cranberry juice	Antibacterial activity.	Green peppers	[22]

* High pressure homogenization: HPH.

**Table 2:** Several methods, experimental conditions, and matrix to obtain nanoemulsions.

Method	Matrix	Experimental Conditions	Structure * and Size (nm)	Ref
High-speed homogenization	Gelatin (0.55 wt%) Thymol (0–0.2 g/mL) in propylene glycol Lecithin (1–2 wt%)	15.000 rpm for 6 min	O/W 137.9 ± 4.5–333.2 ± 7.6	[78]
	Pectin (1–2 wt%) Glycerol (50 wt%) Lemongrass EO (0–1% vol) Tween 80 (0.1% vol)	15.000 rpm for 4 min	O/W 301 ± 4–1222 ± 30	[15]
	Tween 80 (4.5 wt%) *Zataria Multiflora* EO (6 wt%) Cinnamaldehyde (6 wt%)	Droplet addition at 400 rpm for 10 min and 10.000 rpm for 15 min	O/W 93.2–109.1	[79]
High-speed homogenization Ultrasounds	Cinnamon EO 1–5% (*v/v*) Tween 80	5.000 rpm for 10 min, 750 W, 40% amplitude and 10 min	O/W 55.8–120	[49]
High-pressure homogenization	Tween 20 (3.5 wt%) α-tocopherol (3 wt%) Cinnamaldehyde (3% *w/v*) Garlic oil (3% *w/v*) Canoila oil Span 60 (1.5% *w/v*)	5.000 rpm for 5 min, 69–100 MPa and 3 cycles	O/W 111.0 ± 2.0–124.8 ± 1.4	[35]
	Tween 80 (1.25 wt%) Span 80 (3.75 wt%) Rutin (0.1 wt%) Soybean oil	7.000 rpm for 5 min, 100 MPa and 3 cycles	O/W 150	[34]
	Ginger EO (1–5 wt%) Canoila oil Span 60 (4 wt%) Tween 20 (1 wt%)	24.000 rpm for 5 min, 10.000 Psi and 3–6 cycles	O/W 133 ± 5–150 ± 8	[51]
Ultrasounds	Tween 80 (4.5 wt%) *Zataria multiflora* EO (6 wt%)	150 W, 20 KHz, 15 °C and 0–10 min	O/W 90.9–210.5	[50]
	Tween 80 Thymol Medium chain triglycerides	700 W, 40% amplitude, 30 s pulse on/off cycles (0–30 min)	O/W 132.4 ± 2.3	[83]
	Cinnamon oil (2 wt%) 1% Soy protein isolate (98 wt%)	25 kHz, 60% amplitude and 2 min	O/W 141.1	[40]
	Marjoram EO (1 wt%) Tween 80 (30 wt% of EO)	Mix: 3.000 rpm, 200 W, 20 kHz, and 15 min	O/W 97.5	[9]
	Chitosan (2 wt%) Tween 80 (0.2% *w/v*) *Zataria Multiflora Boiss* EO (0.5–1 wt%) *Bunium persicum Boiss* EO (0.5–1 wt%)	50% amplitude, 45 s pulse on and 15 s pulse off (6 min)	O/W 342.3 ± 2.5–506.8 ± 15.8	[58]
Spontaneous emulsification	Curcumin (0.1–0.3 wt%) Tween 80 (9 wt%) Soybean-lecithin (1 wt%) Medium-chain tryglycerides (10%)	Oil phase added dropwise into aqueous phase, 1.000 rpm for 60 min	W/O 126.0 ± 1.5–146.8 ± 0.8	[90]
	Lime EO + corn oil (5% *v/v*) Tween 80 (15% *v/v*)-water (80% *v/v*)	Mix oil phase: 30 min, 750 rpm Add aqueous phase: 30 min, 750 rpm	O/W 21–60	[88]
	2–10% Cinnamon oil + coconut (10 wt%) Tween 80 (10 wt%) Water (80 wt%)	Mix oil phase: 30 min, 750 rpm Add aqueous phase: 30 min, 750 rpm	O/W 81–343	[89]
Point inversion temperature (PIT)	Cinnamon oil (4 wt%) Tween 80 (10–20 wt%) Water (70–80 wt%) Medium-chain tryglycerides (6 wt%)	Mix 30 min at 25 °C, heat 67–78 °C, cool to 15 °C and 4 °C	O/W 23.5 ± 0.8–100.7 ± 1.0	[65]
	Cinnamon bark oil (1%) Tween 80 or 20 (3%) Lauric alginate (0–0.375%)	Mix 15.000 rpm, 30 min at 25 °C, heat 90 °C, 30 min and cool to 4 °C	O/W ~100	[84]
Emulsion inversion point (EIP)	Soybean oil (20%) Tween 80 (25%) Quercetin (0.3%) Glycerol (20%) Water (35%)	Aqueous phase added into oil phase at 12 mL min^−1^. Mix 500 rpm, 30 min	O/W 169 ± 2	[85]

* Oil-in-water (O/W) and water-in-oil (W/O).

**Table 3:** Active coatings based on polysaccharide fro fresh and minimally processed vegetables and fresh-cut fruit.

Material Edible Coating	Functional Ingredient	Benefits	Food	Ref.
Chitosan	Carvacrol	*Escherichia coli* reduction reaching >5 log UCF/g	Cucumber	[127]
Chitosan	*Cinnamomum zeylanicum* EO	Inhibition of *Phytophthora drechsleri*, stored 7 days at 4 °C. Reduction of respiration rates, improving the microbiological quality, preserving the fruit weight.	Cucumber	[133]
Chitosan	Limonene	Prolongation of post-harvest life maintaining weight loss, color, firmness, pH, and organoleptic properties. Reduction of fungal growth	Cucumber	[7]
Quinoa protein and chitosan	Thymol	Cherry tomatoes inoculated with *Botrytis cinereal*. Considerable reduction in fungal growth after 7 days at 5 °C	Cherry tomatoes	[83]
Hydroxypropyl methylcellulose (HPMC), beeswax (BW)	Potassium carbonate, sodium propionate, ammonium carbonate, ammonium phosphate	Reduction of gray mold development on cherry tomatoes. Respiration rate, firmness, sensory flavor, color, off-flavor, and fruit appearance were not badly affected.	Cherry tomatoes	[117]
Chitosan	Chitosan	A 5 days delay in ripening, enhancing the phenolic content and maintaining a low respiration level.	Tomatoes	[134]
Chitosan	Mandarin EO	Inhibition of *Listeria* species. No impact on firmness for 14 days and product color	Green beans	[135]
Chitosan	Mandarin EO	Control the growth of *Listeria innocua.* Reduction in the sample firmness and no color changes during storage.	Green beans	[125]
Chitosan	Bergamot, carvacrol, mandarin and lemon EOs	Inhibition during storage of *Escherichia coli* O157:H7 and *Salmonella Typhimurium* with carvacrol	Green beans	[126]
Pectin	Sesame oil	Antioxidant activity, preservation of quality attributes and control of microbial growth after 12 days.	Cut carrots	[136]
Chitosan	Carvacrol	Control of microbial growth for 13 days at 5 °C.	Cut carrots	[137]
Pectin	Biosecur F440D (citrus extract) and a mixture of four EOs	Increase the shelf-life by 2 days and control of *Listeria* species and *Penicillium chrysogenum*.	Cut carrots	[138]
Maltodextrin and methylcellulose	Lactic acid, citrus extract, lemongrass EO	Inhibition against *Listeria monocytogenes and Escherichia coli.* Variations in the respiration rates with no major color modification.	Cauliflower florets	[139]
Starch and maltodextrin	Lactic acid, citrus extract, lemongrass EO	Inhibition against *Listeria monocytogenes.* No major changes in color, texture and respiration	Cauliflower florets	[140]
Chitosan	Lemongrass EO	Fungal growth was effectively controlled for 21 days at room temperature. Maintenance of the fruit quality: weight loss, firmness, color.	Bell pepper	[141]
Hydroxypropyl methylcellulose	Oregano and bergamot EOs	Reduction in the respiration rate and ethylene production, total weight loss, no surface color change, and total cell count.	Plum	[118]
Carboxymethyl cellulose	Potassium sorbate	Decrease in ripening and minimum changes in the green skin color with no loss of firmness.	Pears	[119]
Alginate	Lemongrass EO	Complete inhibition of the natural microflora for 2 weeks and no significant influence on the quality parameters during storage.	Fuji apples	[128]
Starch and carboxymethyl cellulose	Turmeric EO	Antioxidant activity and low weight loss, firmness loss, and moisture content	Fuji apples	[132]
Chitosan and carboxymethyl cellulose	Citral	Good antimicrobial protection (up to a 5-log reduction), and significant extension of the shelf-life up to 13 days.	Melons	[113]
Chitosan	Lemongrass EO	High inhibition of *Salmonella typhimurium*; inhibition of yeasts, molds, and total mesophilic aerobes. Preservation of total soluble solid content, colour, and antioxidant activity during storage.	Grapes	[120]
Chitosan	Nisin, natamycin, pomegranate and grape seed extract	Antimicrobial effect against yeasts, molds, and mesophilic bacteria.	Strawberries	[142]
Chitosan	Lemon EO	Antifungal activity and no effect on sensorial perception	Strawberries	[121]
Alginate	Carvacrol Methyl cinnamate	Antimicrobial effect. Maintenance of firmness, color retention, and weight loss reduction up to 13 days. Antioxidant effect.	Strawberries	[112]
Alginate Pectin	Eugenol Citral	Antimicrobial and antioxidant effect.	Strawberries	[143]
Chitosan	Bergamot, thyme, and tea tree EOs	Reduction of microbial growth and no changes on the food quality.	Oranges	[122]
Chitosan	*Trans*-cinnamaldehyde Cinnamaldehyde Carvacrol	Reduction of the bacterial and yeasts/molds growth on the fruit. Antimicrobial effect. Shelf-life extension.	Blueberries	[124]
Alginate	Eugenol Citral	Preservation of nutritional and sensory attributes and reduction of microbial spoilage. Antioxidant effect.	*Arbutus unedo L*. fruit	[144]
Alginate	Lemongrass EO	Decrease in the firmness and sensory scores (taste, texture, and overall acceptability). Extension of the shelf-life up to 16 days.	Pineapple	[129]
Alginate and pectin	Eugenol Citral	Antimicrobial and antioxidant effect.	Raspberries	[145]
Pectin	Cinnamon leaf EO	Increase the antioxidant activity, odor acceptability, and inhibition of *Escherichia coli* O157:H7, *Staphylococcus aureus,* and *Listeria monocytogenes.* Preservation of food quality.	Peach	[146]
Pectin	Nisin Calcium chloride Citric acid	Antibrowning effect and maintenance of the sensorial and microbiological quality for more than 9 days.	Persimmon	[130]
Chitosan and pectin	*trans*-cynnamaldehyde-CD inclusion complex	Antimicrobial effect.	Papaya	[147]
Basil seed gum	Oregano EO	Reduction of the microbial population and antioxidant activity.	Apricot	[108]
Pullulan	Calcium chloride Lemon juice	Antibrowning. Enhancement of the overall quality and extension of the shelf-life.	Bananas	[148]

## Data Availability

Not applicable.

## References

[1] Hasan S.M.K.K., Ferrentino G., Scampicchio M. (2020). Nanoemulsion as advanced edible coatings to preserve the quality of fresh-cut fruits and vegetables: A review. Int. J. Food Sci. Technol..

[2] Prakash A., Baskaran R., Paramasivam N., Vadivel V. (2018). Essential oil based nanoemulsions to improve the microbial quality of minimally processed fruits and vegetables: A review. Food Res. Int..

[3] Scarano P., Naviglio D., Prigioniero A., Tartaglia M., Postiglione A., Sciarrillo R., Guarino C. (2020). Sustainability: Obtaining natural dyes from waste matrices using the prickly pear peels of *opuntia ficus-indica (L.) miller*. Agronomy.

[4] Jung J., Deng Z., Zhao Y. (2020). A review of cellulose nanomaterials incorporated fruit coatings with improved barrier property and stability: Principles and applications. J. Food Process Eng..

[5] Palou L., Valencia-Chamorro S.A., Pérez-Gago M.B. (2015). Antifungal edible coatings for fresh citrus fruit: A review. Coatings.

[6] Galus S., Kadzińska J. (2015). Food applications of emulsion-based edible films and coatings. Trends Food Sci. Technol..

[7] Maleki G., Sedaghat N., Woltering E.J., Farhoodi M., Mohebbi M. (2018). Chitosan-limonene coating in combination with modified atmosphere packaging preserve postharvest quality of cucumber during storage. J. Food Meas. Charact..

[8] Ktari N., Feki A., Trabelsi I., Triki M., Maalej H., Slima S.B., Nasri M., Ben Amara I., Ben Salah R. (2017). Structure, functional and antioxidant properties in Tunisian beef sausage of a novel polysaccharide from *Trigonella foenum-graecum* seeds. Int. J. Biol. Macromol..

[9] Almasi H., Azizi S., Amjadi S. (2020). Development and characterization of pectin films activated by nanoemulsion and Pickering emulsion stabilized marjoram (*Origanum majorana L.*) essential oil. Food Hydrocoll..

[10] Liu J., Wang X., Yong H., Kan J., Jin C. (2018). Recent advances in flavonoid-grafted polysaccharides: Synthesis, structural characterization, bioactivities and potential applications. Int. J. Biol. Macromol..

[11] Luo S.Z., Hu X.-F., Jia Y.J., Pan L.H., Zheng Z., Zhao Y.Y., Mu D.-D., Zhong X.Y., Jiang S.-T. (2019). Camellia oil-based oleogels structuring with tea polyphenol-palmitate particles and citrus pectin by emulsion-templated method: Preparation, characterization and potential application. Food Hydrocoll..

[12] Jiang W., Qi J.R., Huang Y., Zhang Y., Yang X.Q. (2020). Emulsifying properties of high methoxyl pectins in binary systems of water-ethanol. Carbohydr. Polym..

[13] Liu C., Tan Y., Xu Y., McCleiments D.J., Wang D. (2019). Formation, characterization, and application of chitosan/pectin-stabilized multilayer emulsions as astaxanthin delivery systems. Int. J. Biol. Macromol..

[14] Cook S.L., Methven L., Parker J.K., Khutoryanskiy V.V. (2018). Polysaccharide food matrices for controlling the release, retention and perception of flavours. Food Hydrocoll..

[15] Mendes J.F., Norcino L.B., Martins H.H.A., Manrich A., Otoni C.G., Carvalho E.E.N., Piccoli R.H., Oliveira J.E., Pinheiro A.C.M., Mattoso L.H.C. (2020). Correlating emulsion characteristics with the properties of active starch films loaded with lemongrass essential oil. Food Hydrocoll..

[16] Kakran M., Antipina M.N. (2014). Emulsion-based techniques for encapsulation in biomedicine, food and personal care. Curr. Opin. Pharmacol..

[17] Karimirad R., Behnamian M., Dezhsetan S. (2019). Application of chitosan nanoparticles containing Cuminum cyminum oil as a delivery system for shelf life extension of Agaricus bisporus. LWT.

[18] Letsididi K.S., Lou Z., Letsididi R., Mohammed K., Maguy B.L. (2018). Antimicrobial and antibiofilm effects of trans-cinnamic acid nanoemulsion and its potential application on lettuce. LWT.

[19] Bhargava K., Conti D.S., da Rocha S.R.P., Zhang Y. (2015). Application of an oregano oil nanoemulsion to the control of foodborne bacteria on fresh lettuce. Food Microbiol..

[20] Donsì F., Cuomo A., Marchese E., Ferrari G. (2014). Infusion of essential oils for food stabilization: Unraveling the role of nanoemulsion-based delivery systems on mass transfer and antimicrobial activity. Innov. Food Sci. Emerg. Technol..

[21] Sow L.C., Tirtawinata F., Yang H., Shao Q., Wang S. (2017). Carvacrol nanoemulsion combined with acid electrolysed water to inactivate bacteria, yeast in vitro and native microflora on shredded cabbages. Food Control.

[22] Maherani B., Harich M., Salmieri S., Lacroix M. (2019). Antibacterial properties of combined non-thermal treatments based on bioactive edible coating, ozonation, and gamma irradiation on ready-to-eat frozen green peppers: Evaluation of their freshness and sensory qualities. Eur. Food Res. Technol..

[23] Gazolu-Rusanova D., Lesov I., Tcholakova S., Denkov N., Ahtchi B. (2020). Food grade nanoemulsions preparation by rotor-stator homogenization. Food Hydrocoll..

[24] Gharibzahedi S.M.T., Hernández-Ortega C., Welti-Chanes J., Putnik P., Barba F.J., Mallikarjunan K., Escobedo-Avellaneda Z., Roohinejad S. (2019). High pressure processing of food-grade emulsion systems: Antimicrobial activity, and effect on the physicochemical properties. Food Hydrocoll..

[25] Solans C., Solé I. (2012). Nano-emulsions: Formation by low-energy methods. Curr. Opin. Colloid Interface Sci..

[26] McClements D.J. (2012). Nanoemulsions versus microemulsions: Terminology, differences, and similarities. Soft Matter.

[27] Li Y., Yokoyama W., Xu S., Zhu S., Ma J., Zhong F. (2017). Formation and stability of W/O microemulsion formed by food grade ingredients and its oral delivery of insulin in mice. J. Funct. Foods.

[28] Ma Q., Zhang Y., Critzer F., Davidson P.M., Zivanovic S., Zhong Q. (2015). Physical, mechanical, and antimicrobial properties of chitosan films with microemulsions of cinnamon bark oil and soybean oil. Food Hydrocoll..

[29] Montes de Oca-Ávalos J.M., Candal R.J., Herrera M.L. (2017). Nanoemulsions: Stability and physical properties. Curr. Opin. Food Sci..

[30] Anton N., Vandamme T.F. (2011). Nano-emulsions and micro-emulsions: Clarifications of the critical differences. Pharm. Res..

[31] Tadros T.F. (2016). Emulsions: Formation, Stability, Industrial Applications.

[32] Gupta A. (2020). Nanoemulsions. Nanoparticles for Biomedical Applications.

[33] Roohinejad S., Greiner R., Oey I., Wen J., Shahin R., Ralf G., Indrawati O., Jingyuan W. (2018). Emulsion-Based Systems for Delivery of Food Active Compounds: Formation, Application, Health and Safety.

[34] Dammak I., de Carvalho R.A., Trindade C.S.F., Lourenço R.V., do Amaral Sobral P.J. (2017). Properties of active gelatin films incorporated with rutin-loaded nanoemulsions. Int. J. Biol. Macromol..

[35] Pérez-Córdoba L.J., Norton I.T., Batchelor H.K., Gkatzionis K., Spyropoulos F., Sobral P.J.A. (2018). Physico-chemical, antimicrobial and antioxidant properties of gelatin-chitosan based films loaded with nanoemulsions encapsulating active compounds. Food Hydrocoll..

[36] Harwansh R.K., Deshmukh R., Rahman M.A. (2019). Nanoemulsion: Promising nanocarrier system for delivery of herbal bioactives. J. Drug Deliv. Sci. Technol..

[37] Chatzidaki M.D., Papadimitriou K., Alexandraki V., Balkiza F., Georgalaki M., Papadimitriou V., Tsakalidou E., Xenakis A. (2018). Reverse micelles as nanocarriers of nisin against foodborne pathogens. Food Chem..

[38] Cheng J., Dudu O.E., Wang D., Li X., Yan T. (2020). Influence of interfacial adsorption of glyceryl monostearate and proteins on fat crystallization behavior and stability of whipped-frozen emulsions. Food Chem..

[39] Jiang T., Liao W., Charcosset C. (2020). Recent advances in encapsulation of curcumin in nanoemulsions: A review of encapsulation technologies, bioaccessibility and applications. Food Res. Int..

[40] Ghani S., Barzegar H., Noshad M., Hojjati M. (2018). The preparation, characterization and in vitro application evaluation of soluble soybean polysaccharide films incorporated with cinnamon essential oil nanoemulsions. Int. J. Biol. Macromol..

[41] Espinosa-Andrews H., Páez-Hernández G. (2020). Optimization of ultrasonication curcumin-hydroxylated lecithin nanoemulsions using response surface methodology. J. Food Sci. Technol..

[42] Abbasi S., Radi M. (2016). Food grade microemulsion systems: Canola oil/lecithin:n-propanol/water. Food Chem..

[43] Amiri-Rigi A., Abbasi S. (2019). Extraction of lycopene using a lecithin-based olive oil microemulsion. Food Chem..

[44] Vélez-Erazo E.M., Bosqui K., Rabelo R.S., Kurozawa L.E., Hubinger M.D. (2020). High internal phase emulsions (HIPE) using pea protein and different polysaccharides as stabilizers. Food Hydrocoll..

[45] Mellinas C., Ramos M., Jiménez A., Garrigós M.C. (2020). Recent Trends in the Use of Pectin from Agro-Waste Residues as a Natural-Based Biopolymer for Food Packaging Applications. Materials.

[46] Wang Y., Zhang A., Wang X., Xu N., Jiang L. (2020). The radiation assisted-Maillard reaction comprehensively improves the freeze-thaw stability of soy protein-stabilized oil-in-water emulsions. Food Hydrocoll..

[47] Wang S., Yang J., Shao G., Qu D., Zhao H., Yang L., Zhu L., He Y., Liu H., Zhu D. (2020). Soy protein isolated-soy hull polysaccharides stabilized O/W emulsion: Effect of polysaccharides concentration on the storage stability and interfacial rheological properties. Food Hydrocoll..

[48] Li Y., Li M., Qi Y., Zheng L., Wu C., Wang Z., Teng F. (2020). Preparation and digestibility of fish oil nanoemulsions stabilized by soybean protein isolate-phosphatidylcholine. Food Hydrocoll..

[49] Frank K., Garcia C.V., Shin G.H., Kim J.T. (2018). Alginate biocomposite films incorporated with cinnamon essential oil nanoemulsions: Physical, mechanical, and antibacterial properties. Int. J. Polym. Sci..

[50] Hashemi Gahruie H., Ziaee E., Eskandari M.H., Hosseini S.M.H. (2017). Characterization of basil seed gum-based edible films incorporated with Zataria multiflora essential oil nanoemulsion. Carbohydr. Polym..

[51] Alexandre E.M.C., Lourenço R.V., Bittante A.M.Q.B., Moraes I.C.F., do Amaral Sobral P.J. (2016). Gelatin-based films reinforced with montmorillonite and activated with nanoemulsion of ginger essential oil for food packaging applications. Food Packag. Shelf Life.

[52] Dammak I., Lourenço R.V., do Amaral Sobral P.J. (2019). Active gelatin films incorporated with Pickering emulsions encapsulating hesperidin: Preparation and physicochemical characterization. J. Food Eng..

[53] Jiménez-Saelices C., Trongsatitkul T., Lourdin D., Capron I. (2020). Chitin Pickering Emulsion for Oil Inclusion in Composite Films. Carbohydr. Polym..

[54] Tyowua A.T., Yiase S.G., Binks B.P. (2017). Double oil-in-oil-in-oil emulsions stabilised solely by particles. J. Colloid Interface Sci..

[55] Xu Y., Chu Y., Feng X., Gao C., Wu D., Cheng W., Meng L., Zhang Y., Tang X. (2020). Effects of zein stabilized clove essential oil Pickering emulsion on the structure and properties of chitosan-based edible films. Int. J. Biol. Macromol..

[56] Yang Y., Fang Z., Chen X., Zhang W., Xie Y., Chen Y., Liu Z., Yuan W. (2017). An overview of pickering emulsions: Solid-particle materials, classification, morphology, and applications. Front. Pharmacol..

[57] Dehghani P., Hosseini S.M.H., Golmakani M.T., Majdinasab M., Esteghlal S. (2018). Shelf-life extension of refrigerated rainbow trout fillets using total Farsi gum-based coatings containing clove and thyme essential oils emulsions. Food Hydrocoll..

[58] Keykhosravy K., Khanzadi S., Hashemi M., Azizzadeh M. (2020). Chitosan-loaded nanoemulsion containing *Zataria Multiflora Boiss* and *Bunium persicum Boiss* essential oils as edible coatings: Its impact on microbial quality of turkey meat and fate of inoculated pathogens. Int. J. Biol. Macromol..

[59] Lamba H., Sathish K., Sabikhi L. (2015). Double emulsions: Emerging delivery system for plant bioactives. Food Bioprocess Technol..

[60] Muschiolik G., Dickinson E. (2017). Double emulsions relevant to food systems: Preparation, stability, and applications. Compr. Rev. Food Sci. Food Saf..

[61] Estévez M., Güell C., De Lamo-Castellví S., Ferrando M. (2019). Encapsulation of grape seed phenolic-rich extract within W/O/W emulsions stabilized with complexed biopolymers: Evaluation of their stability and release. Food Chem..

[62] Liu J., Zhou H., Muriel Mundo J.L., Tan Y., Pham H., McClements D.J. (2020). Fabrication and characterization of W/O/W emulsions with crystalline lipid phase. J. Food Eng..

[63] Huang H., Belwal T., Aalim H., Li L., Lin X., Liu S., Ma C., Li Q., Zou Y., Luo Z. (2019). Protein-polysaccharide complex coated W/O/W emulsion as secondary microcapsule for hydrophilic arbutin and hydrophobic coumaric acid. Food Chem..

[64] Beldengrün Y., Dallaris V., Jaén C., Protat R., Miras J., Calvo M., García-Celma M.J., Esquena J. (2020). Formation and stabilization of multiple water-in-water-in-water (W/W/W) emulsions. Food Hydrocoll..

[65] Chuesiang P., Siripatrawan U., Sanguandeekul R., McClements D.J., McLandsborough L. (2019). Antimicrobial activity of PIT-fabricated cinnamon oil nanoemulsions: Effect of surfactant concentration on morphology of foodborne pathogens. Food Control.

[66] Mitsou E., Pletsa V., Sotiroudis G.T., Panine P., Zoumpanioti M., Xenakis A. (2020). Development of a microemulsion for encapsulation and delivery of gallic acid. The role of chitosan. Colloids Surfaces B Biointerfaces.

[67] Chatzidaki M.D., Balkiza F., Gad E., Alexandraki V., Avramiotis S., Georgalaki M., Papadimitriou V., Tsakalidou E., Papadimitriou K., Xenakis A. (2019). Reverse micelles as nano-carriers of nisin against foodborne pathogens. Part II: The case of essential oils. Food Chem..

[68] Sieniawska E., Świątek Ł., Wota M., Rajtar B., Polz-Dacewicz M. (2019). Microemulsions of essentials oils—Increase of solubility and antioxidant activity or cytotoxicity?. Food Chem. Toxicol..

[69] Karunaratne D.N., Pamunuwa G., Ranatunga U. (2017). Introductory Chapter: Microemulsions. Properties and Uses of Microemulsions.

[70] Acharya D.P., Hartley P.G. (2012). Progress in microemulsion characterization. Curr. Opin. Colloid Interface Sci..

[71] Zhao X., Wang Z. (2019). A pH-sensitive microemulsion-filled gellan gum hydrogel encapsulated apigenin: Characterization and in vitro release kinetics. Colloids Surfaces B Biointerfaces.

[72] Karami A., Kavoosi G., Maggi F. (2019). The emulsion made with essential oil and aromatic water from Oliveria decumbens protects murine macrophages from LPS-induced oxidation and exerts relevant radical scavenging activities. Biocatal. Agric. Biotechnol..

[73] Basak S., Guha P. (2017). Betel leaf (Piper betle L.) essential oil microemulsion: Characterization and antifungal activity on growth, and apparent lag time of Aspergillus flavus in tomato paste. LWT Food Sci. Technol..

[74] Deng L., Taxipalati M., Sun P., Que F., Zhang H. (2015). Phase behavior, microstructural transition, antimicrobial and antioxidant activities of a water-dilutable thymol microemulsion. Colloids Surfaces B Biointerfaces.

[75] Ferreira-Nunes R., da Silva S.M.M., de Souza P.E.N., Magalhães P.d.O., Cunha-Filho M., Gratieri T., Gelfuso G.M. (2018). Incorporation of Eugenia dysenterica extract in microemulsions preserves stability, antioxidant effect and provides enhanced cutaneous permeation. J. Mol. Liq..

[76] Golwala P., Rathod S., Patil R., Joshi A., Ray D., Aswal V.K., Bahadur P., Tiwari S. (2020). Effect of cosurfactant addition on phase behavior and microstructure of a water dilutable microemulsion. Colloids Surfaces B Biointerfaces.

[77] Aswathanarayan J.B., Vittal R.R. (2019). Nanoemulsions and their potential applications in food industry. Front. Sustain. Food Syst..

[78] Li X., Yang X., Deng H., Guo Y., Xue J. (2020). Gelatin films incorporated with thymol nanoemulsions: Physical properties and antimicrobial activities. Int. J. Biol. Macromol..

[79] Amiri E., Aminzare M., Azar H.H., Mehrasbi M.R. (2019). Combined antioxidant and sensory effects of corn starch films with nanoemulsion of Zataria multiflora essential oil fortified with cinnamaldehyde on fresh ground beef patties. Meat Sci..

[80] Yang Y., Marshall-Breton C., Leser M.E., Sher A.A., McClements D.J. (2012). Fabrication of ultrafine edible emulsions: Comparison of high-energy and low-energy homogenization methods. Food Hydrocoll..

[81] Silva E.K., Rosa M.T.M.G., Meireles M.A.A. (2015). Ultrasound-assisted formation of emulsions stabilized by biopolymers. Curr. Opin. Food Sci..

[82] Espitia P.J.P., Fuenmayor C.A., Otoni C.G. (2019). Nanoemulsions: Synthesis, characterization, and application in bio-based active food packaging. Compr. Rev. Food Sci. Food Saf..

[83] Robledo N., Vera P., López L., Yazdani-Pedram M., Tapia C., Abugoch L. (2018). Thymol nanoemulsions incorporated in quinoa protein/chitosan edible films; antifungal effect in cherry tomatoes. Food Chem..

[84] Hilbig J., Ma Q., Davidson P.M., Weiss J., Zhong Q. (2016). Physical and antimicrobial properties of cinnamon bark oil co-nanoemulsified by lauric arginate and Tween 80. Int. J. Food Microbiol..

[85] de Carli C., Moraes-Lovison M., Pinho S.C. (2018). Production, physicochemical stability of quercetin-loaded nanoemulsions and evaluation of antioxidant activity in spreadable chicken pâtés. LWT.

[86] Borrin T.R., Georges E.L., Moraes I.C.F., Pinho S.C. (2016). Curcumin-loaded nanoemulsions produced by the emulsion inversion point (EIP) method: An evaluation of process parameters and physico-chemical stability. J. Food Eng..

[87] Liu Q., Huang H., Chen H., Lin J., Wang Q. (2019). Food-grade nanoemulsions: Preparation, stability and application in encapsulation of bioactive compounds. Molecules.

[88] Liew S.N., Utra U., Alias A.K., Tan T.B., Tan C.P., Yussof N.S. (2020). Physical, morphological and antibacterial properties of lime essential oil nanoemulsions prepared via spontaneous emulsification method. LWT.

[89] Yildirim S.T., Oztop M.H., Soyer Y. (2017). Cinnamon oil nanoemulsions by spontaneous emulsification: Formulation, characterization and antimicrobial activity. LWT Food Sci. Technol..

[90] Nikolic I., Mitsou E., Damjanovic A., Papadimitriou V., Antic-Stankovic J., Stanojevic B., Xenakis A., Savic S. (2020). Curcumin-loaded low-energy nanoemulsions: Linking EPR spectroscopy-analysed microstructure and antioxidant potential with in vitro evaluated biological activity. J. Mol. Liq..

[91] Baiano A. (2014). Recovery of biomolecules from food wastes—A review. Molecules.

[92] Fritsch C., Staebler A., Happel A., Márquez M.A.C., Aguiló-Aguayo I., Abadias M., Gallur M., Cigognini I.M., Montanari A., López M.J. (2017). Processing, valorization and application of bio-waste derived compounds from potato, tomato, olive and cereals: A review. Sustainability.

[93] Mohamed S.A.A., El-Sakhawy M., El-Sakhawy M.A.M. (2020). Polysaccharides, protein and lipid-based natural edible films in food packaging: A review. Carbohydr. Polym..

[94] Falguera V., Quintero J.P., Jiménez A., Muñoz J.A., Ibarz A. (2011). Edible films and coatings: Structures, active functions and trends in their use. Trends Food Sci. Technol..

[95] Parreidt T.S., Müller K., Schmid M. (2018). Alginate-based edible films and coatings for food packaging applications. Foods.

[96] Valencia G.A., Zare E.N., Makvandi P., Gutiérrez T.J. (2019). Self-assembled carbohydrate polymers for food applications: A review. Compr. Rev. Food Sci. Food Saf..

[97] Cazón P., Velazquez G., Ramírez J.A., Vázquez M. (2017). Polysaccharide-based films and coatings for food packaging: A review. Food Hydrocoll..

[98] Jiménez A., Requena R., Vargas M., Atarés L., Chiralt A. (2018). Food Hydrocolloids as Matrices for Edible Packaging Applications.

[99] Pereda M., Amica G., Marcovich N.E. (2012). Development and characterization of edible chitosan/olive oil emulsion films. Carbohydr. Polym..

[100] Pérez-Gago M.B., Rhim J.W. (2013). Edible coating and film materials: Lipid bilayers and lipid emulsions. Innov. Food Packag. Second Ed..

[101] Dhall R.K. (2013). Advances in edible coatings for fresh fruits and vegetables: A review. Crit. Rev. Food Sci. Nutr..

[102] Ghadermazi R., Hamdipour S., Sadeghi K., Ghadermazi R., Khosrowshahi Asl A. (2019). Effect of various additives on the properties of the films and coatings derived from hydroxypropyl methylcellulose—A review. Food Sci. Nutr..

[103] Rozenberga L., Skute M., Belkova L., Sable I., Vikele L., Semjonovs P., Saka M., Ruklisha M., Paegle L. (2016). Characterisation of films and nanopaper obtained from cellulose synthesised by acetic acid bacteria. Carbohydr. Polym..

[104] Priyadarshi R., Rhim J.W. (2020). Chitosan-based biodegradable functional films for food packaging applications. Innov. Food Sci. Emerg. Technol..

[105] Yuan G., Chen X., Li D. (2016). Chitosan films and coatings containing essential oils: The antioxidant and antimicrobial activity, and application in food systems. Food Res. Int..

[106] Aider M. (2010). Chitosan application for active bio-based films production and potential in the food industry: Review. LWT Food Sci. Technol..

[107] Tavassoli-Kafrani E., Shekarchizadeh H., Masoudpour-Behabadi M. (2016). Development of edible films and coatings from alginates and carrageenans. Carbohydr. Polym..

[108] Hashemi S.M.B., Mousavi Khaneghah A., Ghaderi Ghahfarrokhi M., Eş I. (2017). Basil-seed gum containing *Origanum vulgare subsp. viride* essential oil as edible coating for fresh cut apricots. Postharvest Biol. Technol..

[109] Tahir H.E., Xiaobo Z., Mahunu G.K., Arslan M., Abdalhai M., Zhihua L. (2019). Recent developments in gum edible coating applications for fruits and vegetables preservation: A review. Carbohydr. Polym..

[110] Salehi F. (2020). Edible coating of fruits and vegetables using natural gums: A review. Int. J. Fruit Sci..

[111] Ju J., Xie Y., Guo Y., Cheng Y., Qian H., Yao W. (2019). Application of edible coating with essential oil in food preservation. Crit. Rev. Food Sci. Nutr..

[112] Peretto G., Du W.X., Avena-Bustillos R.J., De J., Berrios J., Sambo P., McHugh T.H. (2017). Electrostatic and conventional spraying of alginate-based edible coating with natural antimicrobials for preserving fresh strawberry quality. Food Bioprocess Technol..

[113] Arnon-Rips H., Porat R., Poverenov E. (2019). Enhancement of agricultural produce quality and storability using citral-based edible coatings; the valuable effect of nano-emulsification in a solid-state delivery on fresh-cut melons model. Food Chem..

[114] Andrade R.D., Skurtys O., Osorio F.A. (2012). Atomizing spray systems for application of edible coatings. Compr. Rev. Food Sci. Food Saf..

[115] Chaudhary S., Kumar S., Kumar V., Sharma R. (2020). Chitosan nanoemulsions as advanced edible coatings for fruits and vegetables: Composition, fabrication and developments in last decade. Int. J. Biol. Macromol..

[116] Butnaru E., Stoleru E., Brebu M.A., Darie-Nita R.N., Bargan A., Vasile C. (2019). Chitosan-based bionanocomposite films prepared by emulsion technique for food preservation. Materials.

[117] Fagundes C., Palou L., Monteiro A.R., Pérez-Gago M.B. (2014). Effect of antifungal hydroxypropyl methylcellulose-beeswax edible coatings on gray mold development and quality attributes of cold-stored cherry tomato fruit. Postharvest Biol. Technol..

[118] Choi W.S., Singh S., Lee Y.S. (2016). Characterization of edible film containing essential oils in hydroxypropyl methylcellulose and its effect on quality attributes of “Formosa” plum (*Prunus salicina L*.). LWT Food Sci. Technol..

[119] Kowalczyk D., Kordowska-Wiater M., Zięba E., Baraniak B. (2017). Effect of carboxymethylcellulose/candelilla wax coating containing potassium sorbate on microbiological and physicochemical attributes of pears. Sci. Hortic. (Amsterdam).

[120] Oh Y.A., Oh Y.J., Song A.Y., Won J.S., Song K.B., Min S.C. (2017). Comparison of effectiveness of edible coatings using emulsions containing lemongrass oil of different size droplets on grape berry safety and preservation. LWT.

[121] Perdones A., Escriche I., Chiralt A., Vargas M. (2016). Effect of chitosan-lemon essential oil coatings on volatile profile of strawberries during storage. Food Chem..

[122] Cháfer M., Sánchez-González L., González-Martínez C., Chiralt A. (2012). Fungal decay and shelf life of oranges coated with chitosan and bergamot, thyme, and tea tree essential oils. J. Food Sci..

[123] Nechita P., Roman (Iana-Roman) M. (2020). Review on polysaccharides used in coatings for food packaging papers. Coatings.

[124] Sun X., Narciso J., Wang Z., Ference C., Bai J., Zhou K. (2014). Effects of chitosan-essential oil coatings on safety and quality of fresh blueberries. J. Food Sci..

[125] Severino R., Vu K.D., Donsì F., Salmieri S., Ferrari G., Lacroix M. (2014). Antibacterial and physical effects of modified chitosan based-coating containing nanoemulsion of mandarin essential oil and three non-thermal treatments against Listeria innocua in green beans. Int. J. Food Microbiol..

[126] Severino R., Ferrari G., Vu K.D., Donsì F., Salmieri S., Lacroix M. (2015). Antimicrobial effects of modified chitosan based coating containing nanoemulsion of essential oils, modified atmosphere packaging and gamma irradiation against *Escherichia coli O157:H7* and *Salmonella Typhimurium* on green beans. Food Control.

[127] Taştan Ö., Pataro G., Donsì F., Ferrari G., Baysal T. (2017). Decontamination of fresh-cut cucumber slices by a combination of a modified chitosan coating containing carvacrol nanoemulsions and pulsed light. Int. J. Food Microbiol..

[128] Salvia-Trujillo L., Rojas-Graü M.A., Soliva-Fortuny R., Martín-Belloso O. (2015). Use of antimicrobial nanoemulsions as edible coatings: Impact on safety and quality attributes of fresh-cut fuji apples. Postharvest Biol. Technol..

[129] Azarakhsh N., Osman A., Ghazali H.M., Tan C.P., Mohd Adzahan N. (2014). Lemongrass essential oil incorporated into alginate-based edible coating for shelf-life extension and quality retention of fresh-cut pineapple. Postharvest Biol. Technol..

[130] Sanchís E., Ghidelli C., Sheth C.C., Mateos M., Palou L., Pérez-Gago M.B. (2017). Integration of antimicrobial pectin-based edible coating and active modified atmosphere packaging to preserve the quality and microbial safety of fresh-cut persimmon (Diospyros kaki Thunb. cv. Rojo Brillante). J. Sci. Food Agric..

[131] Oms-Oliu G., Rojas-Graü M.A., González L.A., Varela P., Soliva-Fortuny R., Hernando M.I.H., Munuera I.P., Fiszman S., Martín-Belloso O. (2010). Recent approaches using chemical treatments to preserve quality of fresh-cut fruit: A review. Postharvest Biol. Technol..

[132] Sharif Z.I.M.M., Jai J., Subuki I., Zaki N.A.M.M., Mustapha F.A., Mohd Yusof N. (2019). Characterisation of polysaccharide composite incorporated with turmeric oil for application on fresh-cut apples. J. Phys. Conf. Ser..

[133] Mohammadi A., Hashemi M., Hosseini S.M. (2015). Chitosan nanoparticles loaded with *Cinnamomum zeylanicum* essential oil enhance the shelf life of cucumber during cold storage. Postharvest Biol. Technol..

[134] Mustafa M.A., Ali A., Manickam S., Siddiqui Y. (2014). Ultrasound-assisted chitosan–surfactant nanostructure assemblies: Towards maintaining postharvest quality of tomatoes. Food Bioprocess Technol..

[135] Donsì F., Marchese E., Maresca P., Pataro G., Vu K.D., Salmieri S., Lacroix M., Ferrari G. (2015). Green beans preservation by combination of a modified chitosan based-coating containing nanoemulsion of mandarin essential oil with high pressure or pulsed light processing. Postharvest Biol. Technol..

[136] Ranjitha K., Sudhakar Rao D.V., Shivashankara K.S., Oberoi H.S., Roy T.K., Bharathamma H. (2017). Shelf-life extension and quality retention in fresh-cut carrots coated with pectin. Innov. Food Sci. Emerg. Technol..

[137] Martínez-Hernández G.B., Amodio M.L., Colelli G. (2017). Carvacrol-loaded chitosan nanoparticles maintain quality of fresh-cut carrots. Innov. Food Sci. Emerg. Technol..

[138] Ben-Fadhel Y., Maherani B., Manus J., Salmieri S., Lacroix M. (2020). Physicochemical and microbiological characterization of pectin-based gelled emulsions coating applied on pre-cut carrots. Food Hydrocoll..

[139] Boumail A., Salmieri S., Lacroix M. (2016). Combined effect of antimicrobial coatings, gamma radiation and negative air ionization with ozone on Listeria innocua, Escherichia coli and mesophilic bacteria on ready-to-eat cauliflower florets. Postharvest Biol. Technol..

[140] Boumail A., Salmieri S., St-Yves F., Lauzon M., Lacroix M. (2016). Effect of antimicrobial coatings on microbiological, sensorial and physico-chemical properties of pre-cut cauliflowers. Postharvest Biol. Technol..

[141] Ali A., Noh N.M., Mustafa M.A. (2015). Antimicrobial activity of chitosan enriched with lemongrass oil against anthracnose of bell pepper. Food Packag. Shelf Life.

[142] Duran M., Aday M.S., Zorba N.N.D., Temizkan R., Büyükcan M.B., Caner C. (2016). Potential of antimicrobial active packaging “containing natamycin, nisin, pomegranate and grape seed extract in chitosan coating” to extend shelf life of fresh strawberry. Food Bioprod. Process..

[143] Guerreiro A.C., Gago C.M.L., Faleiro M.L., Miguel M.G.C., Antunes M.D.C. (2015). The use of polysaccharide-based edible coatings enriched with essential oils to improve shelf-life of strawberries. Postharvest Biol. Technol..

[144] Guerreiro A.C., Gago C.M.L., Faleiro M.L., Miguel M.G.C., Antunes M.D.C. (2015). The effect of alginate-based edible coatings enriched with essential oils constituents on *Arbutus unedo* L. fresh fruit storage. Postharvest Biol. Technol..

[145] Guerreiro A.C., Gago C.M.L., Faleiro M.L., Miguel M.G.C., Antunes M.D.C. (2015). Raspberry fresh fruit quality as affected by pectin- and alginate-based edible coatings enriched with essential oils. Sci. Hortic. (Amsterdam).

[146] Ayala-Zavala J.F., Silva-Espinoza B.A., Cruz-Valenzuela M.R., Leyva J.M., Ortega-Ramírez L.A., Carrazco-Lugo D.K., Pérez-Carlón J.J., Melgarejo-Flores B.G., González-Aguilar G.A., Miranda M.R.A. (2013). Pectin-cinnamon leaf oil coatings add antioxidant and antibacterial properties to fresh-cut peach. Flavour Fragr. J..

[147] Brasil I.M., Gomes C., Puerta-Gomez A., Castell-Perez M.E., Moreira R.G. (2012). Polysaccharide-based multilayered antimicrobial edible coating enhances quality of fresh-cut papaya. LWT Food Sci. Technol..

[148] Ganduri V.S.R. (2020). Evaluation of pullulan-based edible active coating methods on *Rastali* and *Chakkarakeli* bananas and their shelf-life extension parameters studies. J. Food Process. Preserv..

[149] Lambert R.J.W., Skandamis P.N., Coote P.J., Nychas G.-J.E. (2001). A study of the minimum inhibitory concentration and mode of action of oregano essential oil, thymol and carvacrol. J. Appl. Microbiol..

[150] Moalemiyan M., Ramaswamy H.S. (2012). Quality retention and shelf-life extension in mediterranean cucumbers coated with a pectin-based film. J. Food Res..

